# Translating COVID-19: From Contagion to Containment

**DOI:** 10.1007/s10912-022-09742-5

**Published:** 2022-06-17

**Authors:** Marta Arnaldi, Eivind Engebretsen, Charles Forsdick

**Affiliations:** 1 Faculty of Medieval and Modern Languages, University of Oxford, Oxford, UK; 2 Faculty of Medicine, University of Oslo, Oslo, Norway; 3 Department of Modern Languages and Cultures, University of Liverpool, Liverpool, UK

**Keywords:** COVID-19, Medical Humanities, Translation, Contagion, Philosophy of Healthcare

## Abstract

This article tests the hypothesis that all pandemics are inherently translational. We argue that translation and translation theory can be fruitfully used to understand and manage epidemics, as they help us explore concepts of infectivity and immunity in terms of cultural and biological resistance. After examining the linkage between translation and coronavirus disease from three different yet interlinked perspectives—cultural, medical, and biocultural—we make a case for a translational medical humanities framework for tackling the multifactorial crisis brought about by the SARS-CoV-2 infection. This innovative entanglement of perspectives has the merit of carving out a new space for translation research at the intersection of the sciences and the humanities, providing sustainable ways to conceptualize the production of science at times of crisis, and challenging conventional views of translation as a primarily linguistic and cultural phenomenon that traditionally does not engage with science.

## Introduction

The outbreak of the coronavirus pandemic has posed a series of translation problems, from the necessity to interpret information for multilingual populations to the need to expeditiously translate laboratory research into a vaccine and medicines for patients (a process itself known as translational medicine). Less obvious translational aspects of the pandemic have also emerged, such as the struggle to find a common language to describe the idiosyncratic experience of contagion and the implications of migration for the spread of the disease. As the boundaries between the private and the collective have collapsed, diverse cultural and linguistic responses have begun to articulate the universality of the coronavirus experience.

The research presented in this article is built upon and tests the assumption that all pandemics are inherently translational. On the one hand, translation provides us with a language to communicate concepts of contagion across languages and cultures; on the other hand, it describes patterns of disease transmission (viruses replicate themselves through translation), culture change (book sales surge, especially of novels read in translation), and community response at a time of crisis (solidarity toward migrant populations and Black, Indigenous, and People of Color [BIPOC]). In a continuous yet imperceptible way, ideas of risk, transmission, and transgression have been attached as much to the transfer of texts as to the spread of diseases. Our immune system fights against outsiders just as national and medical cultures tend to shield themselves from the foreign. From this perspective, it is not coincidental that the word *translation* (to carry across) and the word *contagion* (to touch together) share a common course of action and meaning—namely, that of breaking what “should be joined or joining [what] should be separate” (Douglas 1966, 113). Yet, if we have come to accept that contagion is a “foundational concept in the study of [literature], of religion and of society” (Wald 2008, 2), translation’s epidemiological dimensions have remained, so far, an unexplored dimension of medical humanities.

What role, then, does translation play in the understanding and containment of infectious diseases? What does it mean to translate an illness?^1^ Why is contagion culturally valuable but physiologically destructive? This article addresses these questions in a synchronic way by presenting exploratory research conducted during one of the peaks of the coronavirus crisis (March–June 2020). This original perspective—which we developed through close collaborative work and by integrating different areas of specialism—emerged from the methodological and epistemic intersection of three research projects similarly engaged with translation as a biocultural concept: *Translating COVID-19* (2020), which explored the translational implications of the coronavirus crisis in a series of video interviews; *The Body in Translation: Historicising and Reinventing Medical Humanities and Knowledge Translation* (2017–2020), which aimed to bridge the gap between translation as a humanistic and a medical concept and practice; and *Translating Cultures* (2012–), which examines the role of translation, understood in its broadest sense, in the transmission, interpretation, transformation, and sharing of languages, values, beliefs, histories, and narratives.^2^ As we examined and compared the ways in which translation has been used across these projects’ distinct contexts, a more capacious understanding of the phenomenon crystallized. This understanding led us to the conviction that a translational medical humanities framework has the potential to cross-fertilize notions and experiences of biological and socio-cultural contagion in an interdisciplinary way, thus impacting, and by extension changing, not just the scholarly landscape of medical humanities but also medical knowledge, practice, and policy themselves. This article aspires to suggest how this is the case.

So far, disciplines such as translation studies and translational medicine have attached different literary and metaphorical meanings to the term translation. Here, we seek to understand translation holistically as a powerful source of cross-disciplinary knowledge that should be utilized synergically across both cultural and clinical contexts.^3^ We argue that translation, “no longer deemed a mere instrument of international relations, business, education, and culture” (Apter 2006, 3), plays a key role in the understanding of the epidemiology of diseases, as it helps us explore concepts of infectivity and immunity in terms of cultural and biological resistance. In line with second-wave medical humanities (Whitehead et al. 2017), we urge the adoption of a translational medical humanities framework to evaluate better the mechanisms of biocultural contagion, with science understood as the manifestation of (one, hegemonic) culture rather than the infallible repository of truth (Engebretsen, Henrichsen, and Ødemark 2020). We hope that these initial operations will lay the foundations, on the one hand, for a more capacious understanding of translational medicine that incorporates perspectives from the humanities (especially translation studies) and, on the other, for a paradigmatic shift in translation studies itself as the discipline’s epidemiological implications begin to be revealed.

The article is divided into three parts that examine the nexus between translation and coronavirus disease from three different yet interlinked perspectives: cultural, medical, and biocultural. The translational medical humanities framework invoked here will be elaborated throughout each section before being presented systematically in the final part of the article. The first part provides an overview of the role of translation in the pandemic. It addresses the circulation of medical knowledge across languages and cultures and the linguistic dimensions of (and inequalities evident in) public health messaging. The second part builds on this analysis of wider cultural and linguistic contexts to explore the ways in which the medical concept and practice of translation have been central to the pandemic response. We argue that the spatial and temporal contexts within which scientific knowledge is produced—especially, but not exclusively, at times of emergency—do not account for the *culturality* of medical evidence nor for its translational processes. Drawing upon this investigation, the third and final section of the article makes a case for a translational medical humanities framework for better understanding and dealing with pandemics as the cultural and medical paradigms begin to merge. After demonstrating the commonalities of *contagion* and *translation* within broad frames of inclusion and exclusion, we argue that, in accepting this generalized role of contagion in social and cultural analysis, the previously unexplored epidemiological dimensions of translation become increasingly apparent. By extension, recognizing translation as a biocultural phenomenon that bridges any artificial divide between medicine, on the one hand, and the arts and humanities, on the other, provides new insights into coronavirus epidemiological cases.

## Translation as Multilingual and Multicultural Practice

As we have suggested above, the advent of COVID-19 has revealed what Piotr Blumczynski (2016) dubs the ubiquitous nature of translation, suggesting the extent to which translational processes—often relating to the inter- and intralingual transfer of information but proliferating far beyond these meanings—have been central to medical, social, political, and cultural responses to pandemics. In terms of virology, it is possible to track the rapid first scientific responses to the disease in linguistic terms. Although the Chinese government was criticized for delays in announcing the spread of the new strand of coronavirus in and beyond the city of Wuhan, the response of Chinese scientists was a rapid one. As early as January 2020, researchers at the Shanghai Public Health Clinical Centre & School of Public Health had sequenced and shared details of the initial viral genome on two open-access websites: the National Institutes of Health GENBNK database and virologist.org (Allam 2020, 4). In the same month, they were able to communicate first-hand accounts of the epidemiological and clinical characteristics of the disease in major international journals such as *Nature* (see, e.g., Wu et al. 2020). As Richard Horton, editor of *The Lancet*, noted:

Under immense pressure, as the epidemic exploded around them, they took time to write up their findings in a foreign language and seek publication in a medical journal thousands of miles away. Their rapid and rigorous work was an urgent warning to the world. We owe those scientists enormous thanks. (cited in Kelly 2020)

Central to this dissemination were, therefore, spontaneous acts of translation into English—the dominant language of science—despite the potential *loss* of (crucial) epidemiological information associated with this process. A study in *Nature* suggests that, by the end of January 2020, at least 54 English-language papers on COVID-19 had been published, although it noted that “the search did not include Chinese-language journals” (Storey 2020).^4^

A parallel overview of work published in Chinese in this early period drew on the major national databases (including the China National Knowledge Internet and WANFANG Data) and revealed that, by February 3, 2020, only 23 Chinese-language papers had been published on the virus (Xiang et al. 2020). The production of these articles was clearly governed by a specific research and publication process controlling the translation of scientific findings into Chinese-language papers. However, this imbalance was a cause for some hostility in China, where dissemination in English (as opposed to Chinese) was seen as “a crime against patriotism” and, notably, as the reason for the delayed implementation of effective national measures (Jia 2020). These observations were included in more measured terms in a letter to *The Lancet* by a group of Chinese scientists, who articulated concerns about the unequal distribution of knowledge implicit in this one-way vector of translation and highlighted the potential impediments imposed by this language barrier in terms of the front-line application of findings in China itself.

Many of the research papers about COVID-19 in international journals were written by researchers in China, which led to great concerns because these findings cannot directly benefit frontline health professionals and policy makers because of the language barrier. It is critical for health science to be published in English-language journals to facilitate communication and enable global coordination and timely epidemic response. However, some media were concerned that Chinese researchers within academic organisations concentrated on publishing papers in prestigious international journals but paid inadequate attention to epidemic prevention of COVID-19 and neglected to disseminate their findings within Chinese-language journals. (Xiang et al. 2020, 684)

The writers of the letter indicate the need for dissemination in Chinese (in addition to English) as a solution that allows for both the international circulation of knowledge and its practical application in local epidemiological and clinical situations. They cite the Open Access sharing of Chinese translation of articles in *The Lancet* journals as an instance of best practice.

These early responses to the dissemination and application of scientific knowledge in the initial phases of the COVID-19 outbreak reflect much wider debates about the languages of science and the importance of translation in the circulation of research findings. In a global response to a pandemic, the existence of a lingua franca is essential to permitting a rapid reaction; yet, such scientific monolingualism has implications for the circulation of knowledge (including in terms of impeding crucial counterflows whereby knowledge benefits the linguistic and cultural communities in which it was initially generated).^5^ Translation into (and dissemination in) English may serve as an efficient means of maximizing the global reach of scientific research. However, in the context of knowledge translation (hereafter KT), there is also a need to acknowledge that English is often not considered to be a language among others, with the linguistic, spatial, and cultural limits this implies. Like scientific discourse (see the section *Translation as Medical Concept and Practice* below), English becomes a form of transcendental signifier, a universal language representing a universal truth. In this way, if translation and dissemination are restricted to one language, the localized impact of knowledge can be limited. These restrictions have major implications for the World Health Organization (WHO), a public health body attempting to manage the spread of the pandemic at the global level, and for local communities who are mistrustful of Anglocentric models of disease prevention being imposed on them (which itself can feed into suspicion of vaccines and other health interventions).

Studies of responses to the pandemic in the scientific literature that limit themselves to English-language sources, therefore, significantly underestimate the extent to which the viral proliferation of knowledge needs to be understood in more culturally and linguistically nuanced ways (Nowakowska et al. 2020). Although 85% of articles on COVID-19 may have been published in English-language journals (Taskin et al. 2020), it is important to note that, in certain areas of research and national contexts, there is still much publishing activity in languages other than English.^6^ In the case of COVID-19, however, there does not appear to have been a repetition of the situation in 2004 relating to research on the cross-species transfer of the H5N1 flu to pigs. Discussed initially in Chinese-language literature, the circulation of this work was limited and reached an English-language audience only several months later when one of its authors presented the findings at an international symposium in Beijing on SARS and bird flu.

The COVID-19 pandemic has nevertheless witnessed major changes in the dissemination of scientific research in China, with significant investment in infrastructure, meaning that the crisis has accelerated national reforms of scholarly publishing and, by extension, the sector’s international competitiveness. According to a recent survey, the number of articles on the virus published in Chinese beyond the initial phase of the outbreak marginally exceeds those produced by Chinese scholars in English-language journals (Wang, Xu, and Zhang 2020). It remains clear from the outbreak of COVID-19 that medical research continues to be generated in multilingual, multinational contexts, and the lack of linguistically sensitive approaches to its dissemination not only perpetuates the hegemonies of Anglocentric (and often Anglonormative) patterns of knowledge production^7^ but can also seriously limit its local applicability. In this sense, default monolingualism can be seen as a particular impediment to the translation of knowledge:

The risk is that science is not fully meeting its third mission, which is to inform the public, and this means reaching people in their native languages. It is also unclear which recommendations stem directly from scientific evidence, and which do not; thus, misinformation or vague information circulating via social networks continues to be a threat to public health. (Taskin et al. 2020)

This is why approaches such as those of *The Lancet* journals, which promote linguistic diversity by ensuring the bilingual provision of articles on the virus in both English and Chinese, suggest a growing recognition of the need for post-monolingual understandings, thus foregrounding the importance of translation in knowledge production and dissemination and, in WHO terms, “bridging the language divide in health” (Adams and Fleck 2015).

The question of translation as a means of negotiating multilingualism in scientific research is equally apparent—with different emphases—in relation to public health, where various practices of ideological monolingualism risk limiting the impact of messaging to communities of speakers of non-majority, often minoritized languages (Piller, Zhang, and Li 2020). In a situation where the disproportionate impact of COVID-19 across different ethnic groups has become increasingly apparent, there has been an acknowledgment of the need to understand multilingualism as a key variable in multi-ethnic societies. Translation and interpreting are privileged, as a result, as priorities for any equitable public health policy that seeks to negotiate these linguistic realities. The implications of such linguistic diversity reach beyond disease control and prevention. It is clear, for instance, that language barriers (or the monolingual assumptions that often lead to the limited availability of translation and interpreting) can cause the under-representation of BIPOC groups in COVID-19 studies (Treweek et al. 2020). However, there has been sustained focus, in a wide range of contexts, on the need to factor linguistic considerations into the COVID-19 response, whether these considerations relate to vulnerable mobile populations, such as refugees, or elite travelers, such as concentrations of international tourists (Kluge et al. 2020; Yamawaki 2020). These challenges are equally apparent for more static communities, including Indigenous ones, where COVID-19 has revealed—not least in the United States (US)—that health services are “typically under-resourced with language” (Curtice and Choo 2020, 1753).

Solutions to these situations have included the community-sourcing of translations of COVID-19 related materials, as was the case on a national level with the New Zealand Red Cross (n.d.), and the transnational initiatives of groups such as Translators without Borders (2022), who have responded to the rapid expansion of the virus in linguistically diverse countries. The pandemic has also encouraged the development of further initiatives across languages and national entities in which translation is seen as a means of addressing uneven access to related health information. Guidelines on the impact of the virus on cancer patients, for instance, have been translated into 22 languages in recognition of the fact that, “as most patients worldwide do not speak English, the language of guidance delivery is a major barrier to the dissemination of recommendations in different countries” (Mauri et al. 2020, 759). The pandemic has also led to innovation in digital approaches to translation. Across the African continent, the spread of COVID-19 has been addressed with the rapid expansion of health technologies, central to which have been new functionalities allowing translation of information (on, for example, symptom checkers, testing centers, and emergency contacts) and other COVID-19 messaging into local languages.^8^ At the same time, it is important not to neglect the significance of sign language; in the United Kingdom (UK), the differential provision of interpreters at government health briefings brought to the fore unresolved issues relating to the rights of those whose hearing is impaired.^9^ Routine mask-wearing has also impacted communication for those with hearing loss, and face coverings with see-through panels assist those reliant on lipreading or whose language (such as American Sign Language [ASL]), supplements the use of the hands with facial expressions.

Across these initiatives, the foregrounding of translation has highlighted a set of issues relating to accuracy and effectiveness. According to Rizwan Ahmad (2020), “whether it is the challenge of disseminating correct information or dealing with the challenges of misinformation or fake news about COVID-19, multilingualism must be used as a resource to reach out to the people in languages they understand and trust.” These observations lead, however, to questions about the work of translation and the specific responsibility for this. As Ingrid Piller (2020) notes, a key effect of COVID-19 has been the mainstreaming of debates about linguistic diversity: “In a situation where the wellbeing of everyone depends on that of everyone else, ensuring equitable access to information irrespective of whether someone speaks the state language is in everyone’s best interest.”

Yet, if translation has become central to the fields of emergency linguistics or disaster linguicism,^10^ then the responsibility for its provision also needs to be addressed (Yuming 2020). The UK-based charity Doctors of the World (2021) made available state advice on COVID-19 in 60 different languages. At the same time, they have cautioned against this very approach, underlining the risks of uneven provision and quality should the responsibility not be accepted by central governments and be left instead to local governments and non-profit organizations (Doctors of the World 2020). The challenges of generating and disseminating efficient and effective translations highlight in these ways the crucial intersections of public health and language policy but also reflect the ways in which the latter has often lagged behind the former. These calls for language-sensitive approaches to COVID-19 are consequently part of a wider acknowledgment that translation is central to “the design and delivery of public services and information exchange in multilingual societies,” and “equitable participation in health, education, economic and legal environments relies on freely available and professional language mediation” (Regester and Norton 2018, 160).

The practical role of translation as part of a response to the challenges of multilingualism in the context of COVID-19 is a clear reminder of the wider role of the humanities and social sciences in contexts of health crises, on the one hand, and of the urgency of adopting a translational medical humanities framework, on the other. Anthropologist Melissa Leach, reflecting on parallels between COVID-19 and the Ebola outbreak in West Africa and the Democratic Republic of Congo, notes that “epidemics are fundamentally social phenomena,” meaning that, “whether it is literature, psychology, history or languages, it is to the humanities and social sciences that we turn to make sense of, and escape from, the world around us. Now more than ever, we need the insights, stimulation and comfort they provide” (Leach 2020). It is important to recognize that, while a focus on the management of linguistic diversity provides insight into the social dimensions of virology, epidemiology, and public health, the insights to which Leach refers are multiple, and the role of translation in the therapeutic practices of stimulation and comfort is far from negligible. Translation—as a practice, concept, metaphor, and framework—thus forms a key part of the translational humanities, the pressing need for which has become particularly apparent in the context of COVID-19 (Ostherr 2020). In terms of providing multiple insights, translation is essential. This is the case whether it relates to the generation of key information about the pandemic and its control worldwide (the sharing of this often depends on complex processes of trans-editing), on which the formulation of robust public health policy should depend, or whether it is part of the inclusion of multiple disciplinary perspectives in the formulation of public health emergency measures (a practice now adopted regularly by the WHO in the wake of SARS and Ebola). “Following the science,” with the fetishization of data and evidence this implies, is no longer an effective or adequate response to global health crises such as COVID-19; mono-disciplinary, monolingual thinking is increasingly replaced by the translational practices that underpin interdisciplinary, multilingual working.

## Translation as Medical Concept and Practice

As outlined in the previous section, the COVID-19 crisis is an emblematic demonstration of the importance of translation not only as a multilingual and multicultural practice but also as a medical concept. Translational medicine can be defined as the efficient and effective translation of scientific findings relevant to human disease into knowledge that benefits patients (Littman and Krishna 2011). This definition discloses a neglected reality, as the challenges of translation (as theory and praxis) go to the core of how epidemiological knowledge is used to back various public health measures during the pandemic. In medicine, translation denotes the complex biocultural processes of carrying signals or messages across material, cultural, and semiotic boundaries (Freeman 2009). The term has at least three different meanings: 1) the biological process of creating proteins from an mRNA template; 2) the research procedure of using knowledge from basic research to design therapies, tools, and techniques to address clinical needs; and 3) the health system challenge of making research accessible for clinicians through recommendations, protocols, and technologies (Solomon 2015). All these forms of translation are key to handling the COVID-19 crisis. Understanding and manipulating mRNA translation has been essential to the development of potential vaccine candidates, which are further tested through translational research trials, while the effectiveness of mass public health measures, such as physical distancing or lockdowns, relies strongly on translation into behavioral change.

On the surface, these three forms of medical translation imply three different levels of human involvement, from a process that is allegedly purely biological, via a controlled research setting, to manipulation of everyday human behavior. However, the last three decades of gene engineering research have, of course, clearly demonstrated that gene translations are highly manipulative processes and, more generally, that biological life can be the object and result of biopolitical techniques and strategies (Rose 2001). Similarly, research in science and technology has shown that there is no such thing as a controlled experiment, purified from human manipulation and interpretation (Latour and Woolgar 1986). At the same time, research in evolutionary psychology emphasizes the strong dependence of biological mechanisms on human behavior (Brown and Richerson 2014). However, all these complex entanglements between cultural and biological factors in KT processes have not been adequately theorized in translational medical research until the present moment (Engebretsen, Henrichsen, and Ødemark 2020; Kristeva et al. 2018).

Generally, translation in medicine is conceptualized in terms of a chain consisting of several stages and steps through which research results are gradually prepared for clinical application. Different models distinguish between three or four translational steps, encompassing translation from basic laboratory studies and animal models to controlled trials on humans (T1); translation into large population-based studies, notably randomized controlled trials (T2); translation through systematic reviews and guidelines into clinical recommendations (T3); and translation or implementation into everyday care (T4) (Woolf 2008). The different stages of the translational chain are understood as separate sites of knowledge production and clearly distinguished chronological sequences. Although recent advancements in KT research have increasingly emphasized the need for bidirectional exchange between, for instance, laboratory research and clinical needs, the logic is still based on the assumption that KT takes place through a series of temporally distinguished standardized procedures separated by different sites, such as laboratories, clinical trials, guidelines groups, and everyday healthcare (Engebretsen, Sandset, and Ødemark 2017). Hence, KT implies a clear distinction between evidence as a past presence and its translation as a present presence. At the same time, evidence—when correctly produced—obtains an outside-time status by claiming to be universal. As discussed in the previous section, this transcendental status has traditionally been accompanied by the idea of English as the universal tongue of scientific evidence. Therefore, evidence paradoxically needs to be brought back into time through the act of translation in order to become available for clinical use (Kristeva et al. 2018). KT is thus based on a strictly chronological timeline that ultimately annuls time; as KT dismisses time, further KT steps are needed to bring knowledge back into time again.

This complex chronotopic order is both epistemologically and ethically founded. The validity and credibility of results from laboratory studies, randomized controlled trials, systematic reviews, and clinical guidelines rely strongly on the systematic approach used and the controlled environment under which the studies are performed (Timmermans and Berg 2010). These necessary systematics can only be secured through purification from external, real-world human interference (Wieringa et al. 2017). As such, the scientific space is secluded and shut off from the cultural space. The credibility of such findings is also based on the assumption that the steps are taken in the right order: reviews and guidelines are only considered reliable if they are backed by a sufficient number of previously performed randomized controlled trials; if not, there is simply nothing to translate. During the pandemic, the realization that few experimental studies guided the measures taken caused considerable concerns among KT-oriented policymakers and researchers: as the evidence was not produced in the right order, and based on a strict KT logic, action or translation was not possible. Still, health authorities were required to act.

At the same time, the argument for this structure is fundamentally ethical. In the case of COVID-19, much public attention has been given to the different clinical research phases a vaccine candidate needs to go through to be approved for clinical use. The procedure through which the vaccine candidate is tested on an increasing number of people is considered necessary to ensure that the treatment is effective and safe before it can be offered to entire populations. Although designed for testing pharmaceuticals, this procedure often seems to be used heuristically and underpins the testing and application of *all* health interventions. A good example of this is the discussion about facemasks, where some have advised against their use because there is insufficient evidence to support the claim that masks do not cause harm. Once again, this claim is based on the assumption that evidence is not produced in the right order; as all the translational steps have not been taken and assuming that facemasks are equal to vaccines and drugs, facemasks are potentially harmful. However, as Greenhalgh (2020) has pointed out, this assumption reflects a narrow understanding of the precautionary principle. Although most frequently used to advise caution when implementing interventions that might potentially cause harm, precaution may also be used in the opposite situation, “when serious harm is currently happening and a proposed intervention may reduce that harm” (Greenhalgh 2020, 1072). While drug and vaccine trials invoke the former, other types of health interventions, such as using facemasks, require the latter form of precaution. The problem is that KT logic is based on translation of pharmaceuticals as its implicit model.

The temporal structure implicit in KT also risks undermining the thickness of time involved in both the production and translation of knowledge as well as the interdependence of these two modes of knowing (Kristeva et al. 2018). Both the production and translation of knowledge are complex temporal, material, cultural, and semiotic processes. This requires attention to the technical and social processes at different stages of the translational chain. Several scholars have drawn attention to how experimental settings strongly rely on culturally specific practices and meaning-making and how efforts to undermine and hide these processes are not only unproductive but also potentially dishonest (Stengers 2013). Moreover, although often underestimated, KT processes are also intertextual processes that involve a series of reading responses, the incorporation of texts into new texts, and complex generic dialogues (Kristeva et al. 2018; Bauman 2008). Latour and Woolgar (1986) have famously demonstrated the importance of texts and readings in the laboratory setting. However, their theoretical claim is even more accurate in relation to modern KT practices, such as systematic reviews and guidelines that are entirely based on interpretation and intertextual processing. It is by assessing, summarizing, citing, and paraphrasing other texts that reviews and guidelines come into being. Nevertheless, the whole idea of translating research results—understood as a transcendental signifier—fundamentally undermines this textual and interpretational dimension of the endeavor. We have often heard that more evidence is needed in order to acquire greater certainty about which measures—lockdowns, face masks, school closures, or rather a mitigation strategy to obtain herd immunity—are the most effective to address the pandemic. However, as Greenhalgh (2020) has provocatively argued, we do not need more evidence in many cases. Uncertainty and interpretation are inherent in medical decision-making, and performing more controlled trials to reduce uncertainty and generate more extensive interpretation will not necessarily improve decision-making (Anjum et al. 2018). We need careful and culturally sensitive translation that takes into account the many biocultural facets of translation’s meanings and practical dynamics.

## Theorizing the Translation-Contagion Nexus: Rules and Cures

These wider cultural and medical contexts reveal the extent to which the seemingly unrelated categories of translation and epidemiology interact in complex ways. In this third and last part of the article, we explore how translation and contagion are linked not only semantically and historically but also ontologically and functionally. As it interlaces cultural and medical paradigms, translation provides us with a powerful tool to monitor the evolution of infectious diseases in that it suggests a model to study their spread and a way of envisaging a containment solution.

Even though such a definition of translation may be deemed contradictory—translation being at once disease-causing and disease-stopping—it is in fact justified by translation’s very nature, which is paradoxical by definition. As it functions through and permits the coexistence of opposites—science and culture (Engebretsen, Henrichsen, and Ødemark 2020), dominance and marginality (Venuti 1995), uniqueness and multitude (Reynolds 2020), to give just a few examples—translation positions itself at the intersection of different disciplinary and cultural boundaries, be they professional, spatial, temporal, or linguistic. Ultimately, translation contaminates the supposed purity of an original (scientific) object, multiplies the singular, and stages the unseen workings of transmission, thus enacting a pandemic-like experience.

On the basis of the many meanings the term *translation* can acquire in cultural and medical settings, we have identified three aspects of translation that make it a useful instrument of epidemiological inquiry: temporality, exponentiality, and alterity. We will present them, in turn, with the aim of providing a framework for translational medical humanities research that advances cultural and linguistic views of translation, on the one hand, and that impacts and improves health and well-being, on the other. Even though an analysis of how this framework could inform medical and political interventions falls outside the remit of this study, we hope that the research method proposed here will be developed for and integrated into future discussions around and decisions taken to address epidemiological emergencies.

Homi Bhabha (1994) has distinguished between two forms of temporality that correspond to two forms of existence in and knowledge of the world—namely, the pedagogical and the performative. The former refers to a linear, “continuist [and] accumulative” concept of time, whereas the latter relies on a temporality that is liminal, “repetitious [and] recursive” (Bhabha 1994, 209). From this perspective, the pedagogical-diachronic form correlates with the universal, traditional, and comprehensive understanding of phenomena; it proposes a unified model of time that is heavily dependent on tradition and the past, which, according to our analysis, mirrors the timeframe in which science is produced. Conversely, the performative-synchronic form articulates the complexities of heterogeneity, cultural difference, and fragmentation; with its focus on plurality, diversity, and innovation, it offers a different conception of time that is present-oriented, multiple, and in this sense, inherently translational.

Pandemics offer a privileged observatory from which to examine the ways in which the temporality of translation intersects with the temporality of crises. Bench-to-bedside medicine—despite being a complex cultural construct (see the section *Translation as Medical Concept and Practice* above)—tends to be pedagogical. Yet, scientific research and interventions follow a temporal pattern more likely to be cultural and performative, in which error, risk, and conjecture are as frequent (and valuable) as accuracy, calculus, and conformity. Especially at times of crisis, when people are presented with the urgency of acting in the here and now, our traditional idea of medical evidence—which is produced in the past in order to acquire a status of provability, universality, and truthiness in the present—is doomed to falter. During and after the COVID-19 emergency, the possibility of translating medical research into vaccines, medicines, and public health protocols (in other words, the possibility of producing medical evidence) has been hindered by an allegedly objective and pedagogical understanding of the spatial and temporal contexts within which scientific knowledge is produced. At these times, science, too, reveals itself to be a product of culture. Despite the deep-rooted perception that medical knowledge is universal, acontextual, and non-cultural (Engebretsen, Henrichsen, and Ødemark 2020, 2), the ways in which such knowledge is created are performative and synchronic rather than pedagogical and diachronic. Its translations into the clinical and the political are synchronous, imperfect, and potentially infinite. In this scenario, as Bhabha observes, the present-oriented and fragmentary chronotope implied by notions and practices of translation reflects, supplements, and supports the spatiotemporal settings in which crises erupt (Knight and Stewart 2016).

The second feature that allows us to compare translation and contagion is exponentiality, in that both elements exist because of, and rely on, exponential growth. The coronavirus pandemic has only confirmed this. Initially a medical emergency (an outbreak of pneumonia of unknown etiology) in a specific place (the city of Wuhan in the Hubei province of China) and at a definite time (December 2019), it has become an ongoing economic, political, and racial crisis of global concern. This emergency has triggered a centrifugal conflation of issues that are as epistemological as they are epidemiological. Scientists worldwide have developed complex mathematical modeling that can help us predict and, ideally, flatten the contagion curves. However, even though “technology can still play a major role in modern outbreak analysis … the biggest challenges are often practical rather than computational” (Kucharski 2020, 210). As mathematician Adam Kucharski has observed in *The Rules of Contagion*, a volume that came out during the pandemic, “being able to gather and analyse data is one thing; spotting an outbreak and having the resources to do something about it is quite another” (2020, 210–11). Kucharski goes further by saying that “if we want a better grasp of contagion, we need to account for its dynamic nature. That means tailoring our studies to different outbreaks, moving quickly to ensure our results are as useful as possible, and finding new ways to thread strands of information together” (212); in other words, that means to translate.

Translation’s modus operandi, which is determined by its ontological status, has been recently examined by Matthew Reynolds (2020). Translation, Reynolds argues, is not a channel but a prism; it does not reproduce but proliferates. In this sense, we need

to see translation, not as fundamentally a single act involving one source-text in one language, and one translation-text in one another language, which just happens to occur again and again, but rather as paradigmatically generating multiple texts, so that “translation” becomes the process of turning from one language into others, da una lingua in altre, producing chains of signifiers in target languages, creating multiple equivalent, authentic texts, while “a translation” correspondingly figures as just one of many actual and/or possible linguistic realisations. (Reynolds 2020, 2)

Reynolds’ words, which refer to the afterlives of texts, can in fact be read as the account of a biological contagion.

As a matter of fact, ideas and feelings multiply as exponentially as diseases to the point that the concurrent “spread of any number of insidious, malevolent forces” (Ostherr 2002, 1), from viruses to racism to paranoia, is more consistent than it might first appear. As health and media scholar Kirsten Ostherr has pointed out, the linkage of disease and difference has a long history, as the invisibility of contagion is inevitably implicated with “other potentially invisible aspects of identity, particularly race and sexuality, in an effort to pin the elusive contaminant to a concrete embodiment of ‘otherness’” (2002, 1–2). Even though the “development of microbiology [in the late nineteenth century] enabled newly successful forms of treatment and prevention of contagious diseases based on laboratory identification of disease-causing microorganisms,” the discourse of contagion has continued “to locate disease at the borders of the normative (white, literate, propertied, male) national body” in a way that has favored the dissemination of conspiracy theories often constructed on the fear of foreign or alien invaders (Ostherr 2002, 7–8).

Alterity—be it represented by a female, homosexual, immigrant, or not-human body, often referred to as an enemy—is inherently pathogenic, as it challenges and penetrates the sexual, cultural, and geopolitical borders that have been set up by individuals, institutions, and nations. Ostherr’s insightful reading juxtaposes a “global transportation network,” in which bodies and commodities circulate, with a “global disease network,” in which “the threat posed by internal exchange … resides in the potentially undetected passage of invisible contaminants across institutionally regulated borders” (Ostherr 2002, 2, 12). It is not coincidental that the outbreak of the coronavirus pandemic has coincided with an increase in xenophobic episodes—beginning with former US President Donald Trump’s definition of the virus as a foreign enemy and culminating in the killing of African American citizen George Floyd at the hands of a white police officer in Minneapolis on May 25, 2020.^11^

So far, we have considered translation’s pathogenicity, whose patterns of replicability have proven to be comparable to those of a virus. The DNA of translation, however, contains information not only about illness but also about cure, as shown in the first instance, by its present-oriented temporality. The coronavirus crisis, which has resulted from a combination of biological, cultural, and environmental forces, offers once more a paradoxical case in point.^12^

It has become increasingly evident that a treatment for coronavirus disease cannot be exclusively pharmacological (Sandset, Heggen, and Engebretsen 2020). Even though “none of the ten highest COVID-19 case-notifying countries reported data related to ethnicity,” Black, Asian, and minority ethnic people have, as we note above, suffered from the disease disproportionately (Pareek et al. 2020, 1421). Ethnicity, which is “a complex entity composed of genetic make-up, social constructs, cultural identity, and behavioural patterns, … could [in fact] interplay with virus spread through cultural, behavioural, and societal differences including lower socioeconomic status, health-seeking behaviour, and intergenerational cohabitation” (Pareek et al. 2020, 1421–22). On the basis of these observations, Pareek et al. (2020, 1422) have concluded that “if ethnicity is found to be associated with adverse COVID-19 outcomes, this must directly, and urgently inform public health interventions globally.”

On the basis of these premises, we believe that the use of a translational medical humanities framework during and after the coronavirus emergency could help facilitate inter- and cross-cultural communication, on the one hand, and mediate among different epistemic discourses (namely, the cultural and the clinical), on the other, with the effect of mobilizing, rather than segregating, knowledge. The science-humanities framework put forward in this article has the potential to contribute to the emerging fields of crisis translation, emergency linguistics, and disaster linguistics by re-defining the role of translation at times of social upheaval, not just as a vector of communication amongst speakers of different languages but also as a bridge-concept between the seemingly discordant speeches of science and the humanities.

We expect that a translational medical humanities framework will permit scientific and cultural notions of contagion to cross-fertilize each other while also facilitating the interpolation of biological and cultural factors. This is possible on two counts: because translation celebrates rather than condemns difference (Venuti 1998; Ricoeur 2006) and because translation is a “language instinct” (Pinker 1994, 16) that contributes to our survival as a fundamentally social and talking species. In both cases, translation provides paradigms of hospitality through which we can relate to otherness in all its forms—ethnic, cultural, and biological.

## Conclusions

The article has put forward a biocultural understanding of translation that can help us confront and potentially contain epidemiological outbreaks, such as the crisis brought about by coronavirus disease. We have compared and interwoven notions and practices of translation in the sciences and the humanities in order to investigate the consequences and mutual benefits of this reciprocal engagement. This innovative entanglement of perspectives has the merit of 1) carving out a new space for translation research at the intersection of the sciences and the humanities (interdisciplinarity being translational by definition); 2) providing sustainable ways of conceptualizing the production of science at times of crisis when the interpretation of existing results is perhaps more urgent than the generation of evidence-based ones; and 3) challenging and transforming conventional views of translation as a primarily linguistic and cultural phenomenon that traditionally does not engage with science.

We hope that the preliminary steps undertaken here can pave the way for future interdisciplinary inquiries into translation as an epistemic framework suited not only for crossing linguistic, cultural, and disciplinary boundaries at times of emergency but also for shaping and impacting medical knowledge, practice, and policy. As the coronavirus pandemic has revealed to us, the rules of health and contagion rely upon a delicate balance of factors that science on its own can neither explain nor re-establish.

## Data Availability

Not applicable. This study does not use custom code or mathematical algorithms.

## Footnotes

1 In this article, we do not distinguish between concepts of disease (a pathology) and illness (the emotional, relational, and social impact of diseases). Even though COVID-19 falls under the category of disease, its cultural and social implications have rapidly turned it into an illness (Eisenberg 1977).

2 Details on these projects’ funding, rationale, and aims can be found by visiting the projects’ webpages: *Translating COVID-19*, led by Marta Arnaldi (https://www.queens.ox.ac.uk/translating-covid-19); *The Body in Translation*: *Historicising and Reinventing Medical Humanities and Knowledge Translation*, led by Eivind Engebretsen and John Ødemark (https://www.med.uio.no/helsam/english/research/projects/body-in-translation/); and *Translating Cultures*, led by Charles Forsdick (http://translatingcultures.org.uk).

3 For a discussion of translation’s epistemic potential across different disciplines and systems of thought, see Marais (2019).

4 Hacking (2003) provides an illuminating, philologically inflected study of the mutual implications among collective risk, the words we use, and the harm we/they may cause. We thank one of the anonymous peer reviewers for suggesting this important reference.

5 On English as the global language of science, see Gordin (2015) and Montgomery (2013).

6 A recent survey of over 75,000 scientific documents on biodiversity conservation published in 2014 revealed that over a third were not published in English (see, Amano, González-Varo, and Sutherland 2016). Also, scientists in Brazil currently publish around 50,000 articles a year, of which about 60% are in Portuguese (see, Meneghini and Packer 2007).

7 As Jinghai Li (2020) notes, “the opinions and papers of some individuals are more easily published and circulated than those of others; the people at a disadvantage include junior scientists, researchers from scientifically underdeveloped countries and those from non-English-speaking countries.”

8 The trilingual site covidguide.africa, for example, provides information in English, Igbo, and Yoruba (for further discussion, see Adepoju [2020]).

9 Interpreters were employed in Wales and Scotland, where British Sign Language (BSL) is a legally protected language, but not in England. In Northern Ireland, two sign language interpreters were routinely present at press conferences for Irish Sign Language as well as BSL.

10 These are new areas that foreground the importance of language and communication in contexts of pandemics and address the ways in which linguistic minorities are particularly vulnerable in such situations as a result of language-based discrimination.

11 This is evident in Donald Trump’s words delivered as part of his Oval Office Address on 11 March 2020: “This is the most aggressive and comprehensive effort to confront a foreign virus in modern history.” For a discussion of the linkage between epidemics and xenophobia, see Gilman (2021).

12 On the relation between environmental issues and COVID-19, see, for instance, Rume and Didar-UI Islam (2020).

## Declarations

### Data transparency

The authors affirm that this manuscript is an honest, accurate, and transparent account of the study being reported and that no important aspects of the study have been omitted.
